# Stress and movement trend of lower incisors with different IMPA intruded by clear aligner: a three-dimensional finite element analysis

**DOI:** 10.1186/s40510-023-00454-7

**Published:** 2023-02-13

**Authors:** Yixin Li, Shengzhao Xiao, Yu Jin, Cheng Zhu, Ruomei Li, Yikan Zheng, Rongjing Chen, Lunguo Xia, Bing Fang

**Affiliations:** 1 Department of Orthodontics, Shanghai Ninth People’s Hospital, Shanghai Jiao Tong University School of Medicine;College of Stomatology, Shanghai Jiao Tong University; National Center for Stomatology; National Clinical Research Center for Oral Diseases; Shanghai Key Laboratory of Stomatology; Shanghai Research Institute of Stomatology, 500 Quxi Road, Shanghai, 200011 China; 2 Translational Medicine Research Platform of Oral Biomechanics and Artificial Intelligence, Department of Orthodontics, Shanghai Ninth People’s Hospital, Shanghai Jiao Tong University School of Medicine;College of Stomatology, Shanghai Jiao Tong University; National Center for Stomatology; National Clinical Research Center for Oral Diseases; Shanghai Key Laboratory of Stomatology; Shanghai Research Institute of Stomatology, Shanghai, 200011 China

## Abstract

**Background:**

During the intrusion of lower incisors with clear aligners (CAs), root disengagement from the alveolar bone often occurs, resulting in serious complications. This study aimed to determine the potential force mechanism of the mandibular anterior teeth under the pressure of CA, providing theoretical data for clinical practice.

**Methods:**

In this study, a 3D finite element model was established, including the CA, periodontal ligament, and mandibular dentition. Incisor mandibular plane angles were set as 5 groups: 90°, 95°, 100°, 105°, and 110°. The 4 mandibular incisors were intruded by 0.2 mm, while the canines were the anchorage teeth. The stress, force systems, and potential movement trends of mandibular anterior teeth were obtained.

**Results:**

The compressive stress of the incisors was concentrated in the lingual fossa, incisal ridge, and apex. With the increase in IMPA, the moment of central incisors changed from lingual crown moment to labial crown moment, with the turning point between 100° and 105°, but the center of resistance (CR) was always subjected to the force toward the lingual and intrusive direction. The force and moment toward the labial side of the lateral incisors were greater than those toward the central incisors. The canines always tipped distally and received extrusive force with no relationship with IMPA.

**Conclusions:**

With the increase in the initial IMPA, the direction of labiolingual force on the mandibular incisors was reversed. However, the root of the lower incisors always tipped labially, which indicated fenestration and dehiscence.

## Background

Class II malocclusion is accompanied by symptoms such as convexity, mandibular retraction, deep overbite, and deep overjet, which lead to an imbalance of facial esthetics and adversely affect physical and mental health. In clinical practice, it is necessary to prioritize leveling the curve of Spee (COS). The normal COS creates conditions for the retraction of maxillary anterior teeth, thus reconstructing facial esthetics. The level of COS directly affects the automatic rotation of the mandible and the change in the esthetic ratio. Therefore, the intrusion of mandibular incisors is the key step for the treatment of Class II malocclusion.

**Fig. 1:**
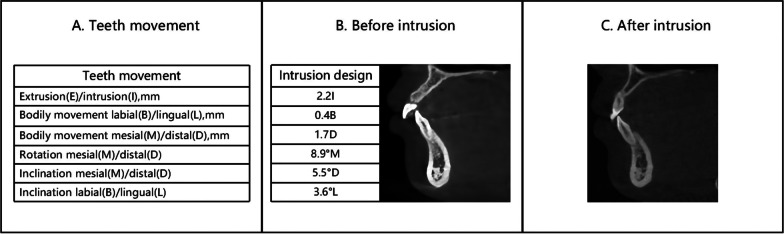
Changes in the relationship between the root and the alveolar bone during the intrusion of mandibular incisors. Rectangular attachments were set on the labial surface on the lower canines. Four mandibular incisors were simultaneously intruded. The CBCT images of the mandibular left lateral incisor are displayed. **A** The movement data from top to bottom are extrusion (E) or intrusion (I), labial movement (B) or lingual movement (L), mesial movement (M) or distal movement (D), mesial rotation (M) or distal rotation (D), mesial inclination (M) or distal inclination (D), labial inclination (B) or lingual inclination (L). **B** Before intrusion, although the alveolar bone on the root labial surface was thin, the root was located in the alveolar bone. The designed direction of intrusion was basically along the tooth axis. **C** After the intrusion of 2.2 mm, the root moved to the labial side and separated from the alveolar bone

For different classifications and severities of Class II malocclusion, IMPA also varies. The goal of the first stage of treatment is to open the occlusion and level the COS. Currently, clear aligner technology is widely used in clinical practice. Compared with fixed appliances, CAs are more esthetic and comfortable [1]. However, during the intrusion of the lower incisors, CA is not effective enough, when accompanied by labial alveolar bone damage (Fig. 1). Kravitz et al. [2] reported that the accuracy of the CA in the intrusion of the mandibular central and lateral incisors was 44% and 45%, respectively. The study of Al-Balaa et al. [3] suggested that the accuracy of intrusion was 44.71% in lower incisors.

Many researchers [4–8] have performed biomechanical studies on CA by three-dimensional (3D) FE analysis. In previous studies, the mandibular incisors in the models were all upright. To explore the actual efficiency of the invisible appliance on lower anterior teeth, Ni et al. [4] established a 3D FE model by scanning the normal occlusion mandible with cone-beam computed tomography (CBCT). In the studies of Cortona et al., Yokoi et al., and Gabriele et al. [6–8], instead of real mechanical parameters and geometry, the CA was set as a homogeneous solid body, expanding uniformly along the outer surface of the crown. Previous studies have not explained the common risk of fenestration and dehiscence in the intrusion of mandibular incisors. Therefore, it is necessary to further explore the force mechanism of intrusion with CA in Class II malocclusion.

According to the compensation characteristics of the mandibular incisors in Class II malocclusion, this study intended to establish 3D FE models including different initial IMPA incisors, in which the incisors are 2 mm above the occlusal plane. By analyzing models, the potential force mechanism of mandibular incisor intrusion can be obtained to guide the safe application of CA in clinical practice.

## Materials and methods

### Establishment of the 3D FE model

A standard mandibular model (i21D-400C, Nissin Dental Products Inc., Tokyo, Japan) was scanned by industrial CT (METROTOM 1500, Carl Zeiss AG, Oberkochen, Germany) with a slice of 0.1 mm. The obtained images were imported into Mimics 17.0 (Materialise NV, Leuven, Belgium) for 3D reconstruction. HyperMesh 14.0 (Altair Engineering Inc., Troy, USA) was used to further process and finally obtain the 3D FE model of the mandibular dentition. The PDL was set to extend uniformly on the root by 0.3 mm [9]. Based on Masterforce (Angelalign Technology Inc., Shanghai, China), the thermoforming process of CA was simulated to obtain authentic nonuniform thickness data.

A world coordinate system in engineering was applied in the research. The *X*-axis is the mesiodistal axis (the mesial direction is positive, and the distal direction is negative). The *Y*-axis is the labiolingual axis (the labial direction is positive and the lingual is negative). The *Z*-axis is the vertical axis (intrusion is positive and extrusion is negative).

Five groups of FE models were established by the above method. The depth of COS was 3.0 mm. The initial IMPA of the mandibular incisors were 90° (25° in the world coordinate system), 95° (30° in the world coordinate system), 100° (35° in the world coordinate system), 105° (40° in the world coordinate system), and 110° (45° in the world coordinate system).

The teeth were divided into tetrahedral elements [10], the PDLs into hexahedral elements, and the aligners into quadrilateral shell elements with true thickness information. The number of elements for the dentition, PDL, and the aligner was identically applied as 96, 168, 308, 219, and 6530, respectively.

### Material properties and boundary conditions

The teeth were set as a continuous, homogeneous, and uniform linear elastic material with an elastic modulus of 20,600. CA is a linear elastic material with a nonuniform thickness. The material properties of MC-S widely used in the clinic were adopted for CA. The PDL was set as a viscoelastic material [11] (Table 1).

**Table 1:** Material properties

	Young’s modulus (MPa)	Poisson’s ratio
Tooth	20,600	0.30
PDL	0.689	0.49
Aligner	1000	0.40

The root and the PDL were joined by bonded contact, that is, they shared nodes and did not move relatively [12]. The crown and the CA were constrained by frictional contact, and the frictional coefficient was 0.3.

### Intrusion design and outcomes

**Fig. 2:**
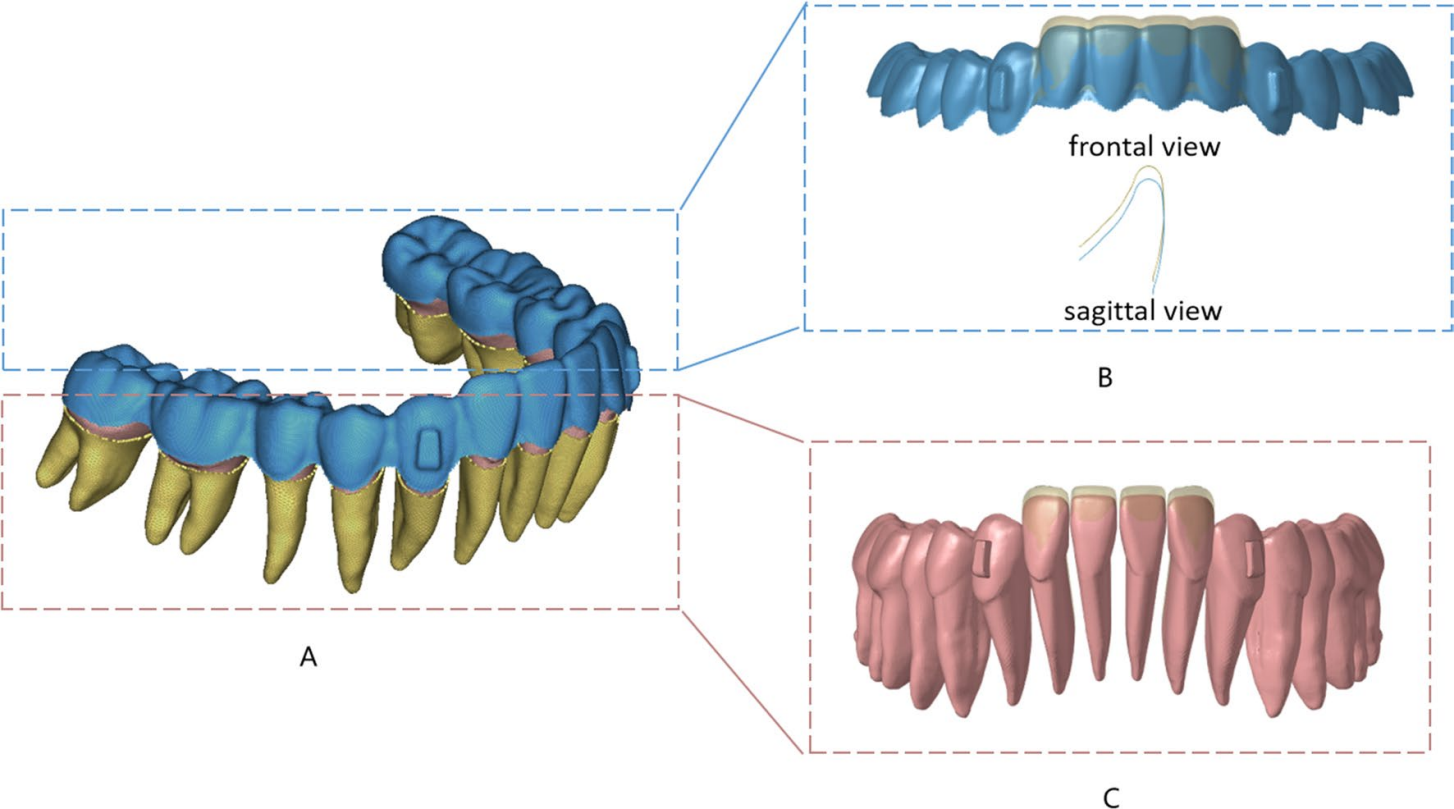
FE model and intrusion design. **A** CA including rectangular attachments on the labial surfaces of mandibular canines (blue), teeth (pink), PDL (yellow). **B** Aligner before (yellow) and after (blue) the mandibular incisors were simultaneously intruded by 0.2 mm. **C** Dentition before (yellow) and after (pink) the mandibular incisors were simultaneously intruded by 0.2 mm

For five FE models with a COS depth of 3.0 mm and initial IMPA of 90°, 95°, 100°, 105°, and 110°, the mandibular incisors were simultaneously intruded by 0.2 mm. According to the common clinical design, rectangular attachments were set on the labial surface on canines (Fig. 2). Referring to the Tweed analysis, when the IMPA is 90°, it is a well-balanced face [13]. Therefore, the intrusion direction was consistent in the five groups of models, that was, along the long axis of the incisor when the IMPA was 90°. The stress of teeth and PDLs, the moment, the force of the CR, and the potential movement trend of lower anterior teeth were obtained.

The von Mises stress, minimum principal stress, and maximum principal stress were calculated. von Mises stress reflects the overall stress concentration without direction. The maximum principal stress reflects the tensile stress, and the minimum principal stress reflects the compressive stress.

## Results

**Fig. 3:**
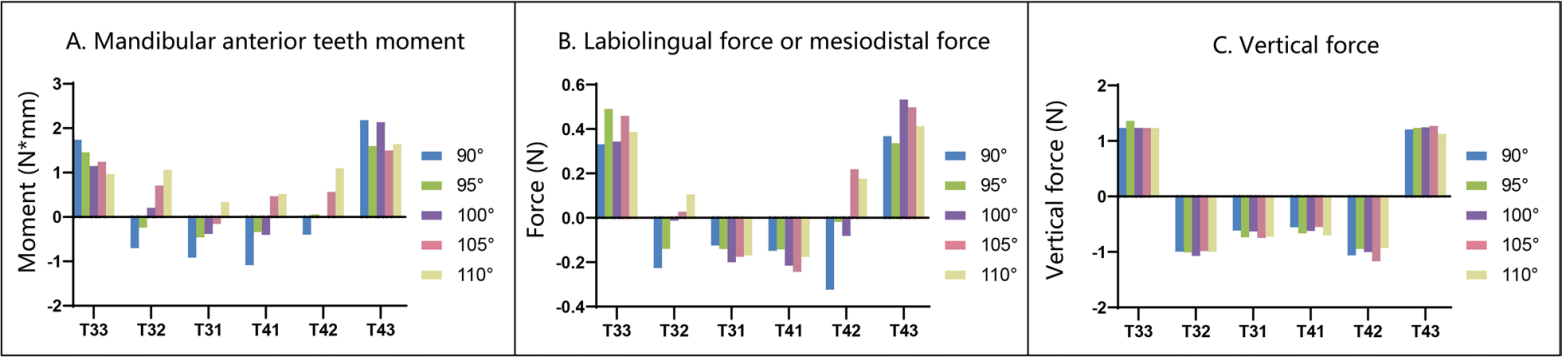
Comparison of the force pattern of the bilateral identical teeth. Mandibular bilateral identical teeth were symmetrical in moment, mesiodistal force, labiolingual force, and vertical force. **A**. The mandibular anterior tooth moment: The mandibular left canine (33) and mandibular right canine (43) received distal crown moment basically without difference. The labial crown moment of the mandibular left central incisor (31), mandibular right central incisor (41), mandibular left lateral incisor (32), and mandibular right lateral incisor (42) were symmetrical. **B**. The labiolingual or mesiodistal force: CR of 33, 43 received force toward mesial direction without difference. 31, 32, 41, and 42 received labiolingual force without difference. **C**. The vertical force: 33 and 43 were subjected to the extrusive force without difference. 31, 32, 41, and 42 were subjected to the intrusive force without difference

The force patterns of the bilateral identical teeth were basically symmetrical (Fig. 3). Therefore, in the subsequent analyses, the mandibular left incisors and canine will be used to represent the stress distribution and potential movement trend of the bilateral identical teeth. In the study, the force of the CR was used to represent the resultant force on the tooth (including the crown and root). Since both incisors and canines are single-rooted teeth, the CR was 2/5 away from the alveolar ridge and 3/5 away from the root apex [14].

### Force systems and stress of incisors

**Fig. 4:**
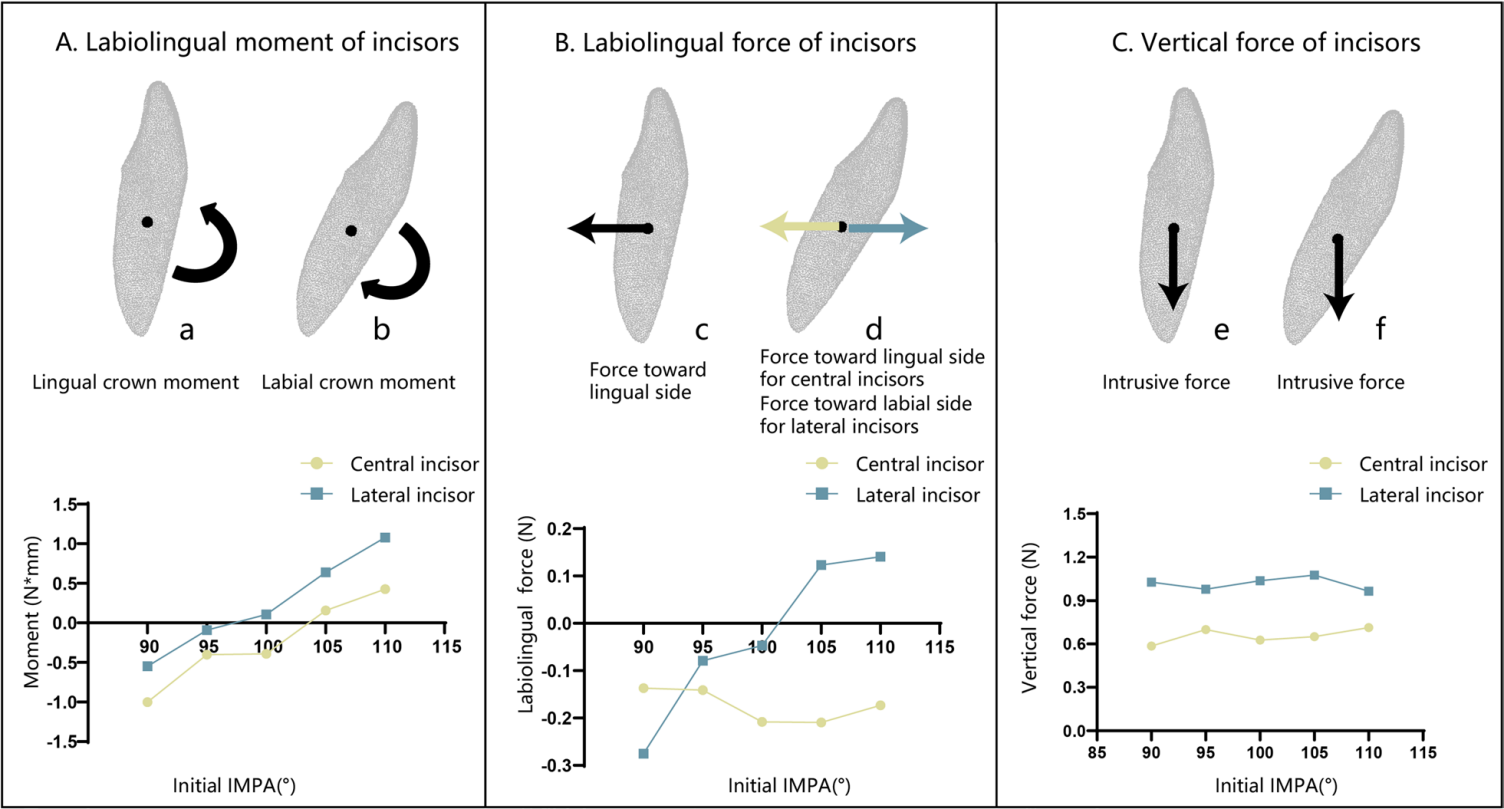
Force system analysis of incisors with different initial IMPA during the intrusion. The *X*-axis is the initial IMPA, and the *Y*-axis is the moment or force. The black dot in the figure is the CR. The black arrow represents the same direction of force or moment on the central incisor and the lateral incisor. The yellow arrow represents the direction of force or moment on the central incisor, and the blue arrow represents that on the lateral incisor. **A**. Labiolingual moment of incisors: a is the moment of the incisor at 90° (lingual crown moment); b is the moment of the incisor at 110° (labial crown moment). The positive value represents the labial crown moment. When the initial IMPA was less than or equal to 100°, central incisors were subjected to lingual crown moment, and when it was larger than or equal to105°, they were subjected to labial crown moment. When the IMPA was less than or equal to 95°, lateral incisors were subjected to lingual crown moment. When IMPA was greater than or equal to 100°, the moment transformed into labial crown moment for lateral incisors. **B**. Labiolingual force of incisors: c is the force direction of the incisors at 90° (force toward lingual direction); *d* is the force direction of the incisors at 110° (force toward lingual direction for central incisors, force toward labial direction for lateral incisors). The positive value represents the force toward the labial direction. The CR of the central incisors was always subjected to force in the lingual direction, and the force value did not change significantly with IMPA. For lateral incisors, when the IMPA was less than or equal to 100°, the CRs of the lateral incisors were subjected to a force in the lingual direction. When it was greater than or equal to 105°, they were subjected to the force toward the labial direction. **C**. Vertical force of incisors: e is the force direction of the incisors at 90° (intrusive force); f is the force direction of the incisors at 110° (intrusive force). A positive value represents intrusion. The central incisors and lateral incisors were always subjected to vertical intrusive force, and the force value did not change significantly with increasing IMPA. The intrusive force of the lateral incisors was always greater than that of the central incisors

When the initial IMPA of the central incisor was 90°, lingual crown moment was applied during the intrusion with CA. With the increase in the initial IMPA, the lingual crown moment decreased and gradually transformed into the labial crown moment. When the initial IMPA reached 105°, the central incisor was subjected to labial crown moment. The CR was always subjected to the force toward the lingual and intrusive directions with the increase in the initial IMPA, and the magnitude of the intrusive force did not change significantly (Fig. 4).

The force system of lateral incisors was similar to that of central incisors, but the labial crown moment and intrusive force were always greater than those of central incisors. When the initial IMPA was less than or equal to 95°, the lateral incisor was subjected to lingual crown moment, and the CR received a resultant force toward the lingual side and a force in the vertical intrusive direction. When the initial IMPA reached 100°, the direction of labiolingual force and vertical force remained unchanged, but the moment changed to the labial crown moment. When the initial IMPA was greater than or equal to 105°, the moment changed to the labial crown moment, and the CR was subjected to the force toward the labial direction (Fig. 4). The results revealed that CA produced the intrusive force of mandibular incisors through structural deformation. The deformation of the CA structure mainly occurred in the relative change of the incisal ridge and the gingival margin. In addition to intrusive forces, CA structural deformation also generated unwanted forces that may cause unwanted potential movement trends of the lower incisors during treatment.

**Fig. 5:**
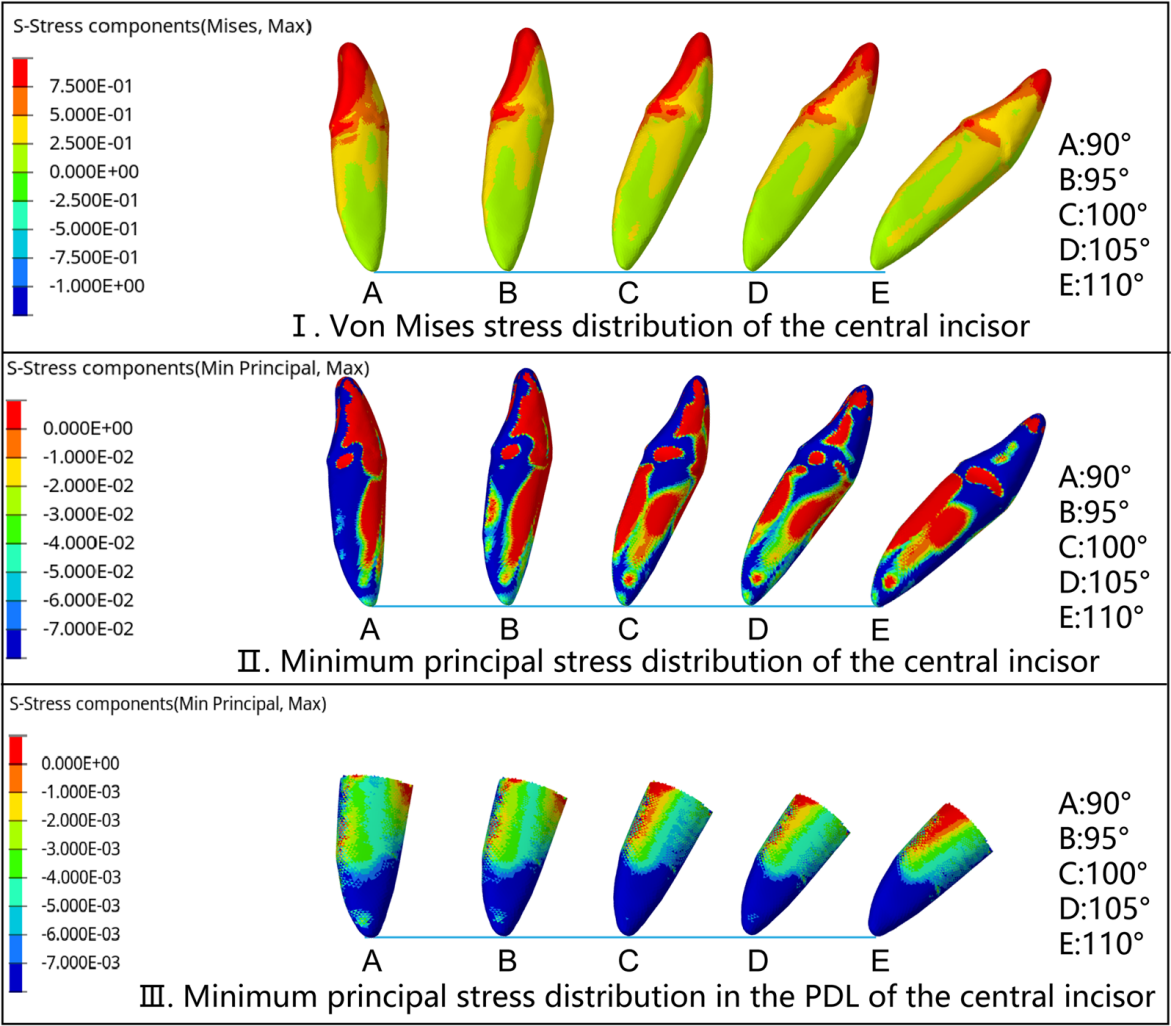
Stress distribution of central incisors with different IMPA values during the intrusion by 0.2 mm. The sequence is A (90°), B (95°), C (100°), D (105°), and E (110°). **I**. von Mises stress distribution of the central incisor: The crown stress was mainly concentrated in the lingual fossa and incisal ridge, and the root stress was concentrated in the lingual surface and the proximal cervical area. **II**. The minimum principal stress distribution of the central incisor: The compressive stress of the crown was concentrated in the lingual fossa and the incisal ridge. The compressive stress on the root was concentrated in the lingual surface and root apex when the initial IMPA was 90° and 95°. With the increase in IMPA, the root compressive stress area changed to the labial surface. When the IMPA was 110°, the compressive stress was concentrated in all areas of the root labial surfaces, from the apex to the root cervix. **III**. The minimum principal stress distribution in the PDL of the central incisor: The compressive stress was mainly concentrated in the apex. With the increase in IMPA, the compressive stress concentration area expanded to the labial middle 1/3 and cervical 1/3 of the PDL

**Fig. 6:**
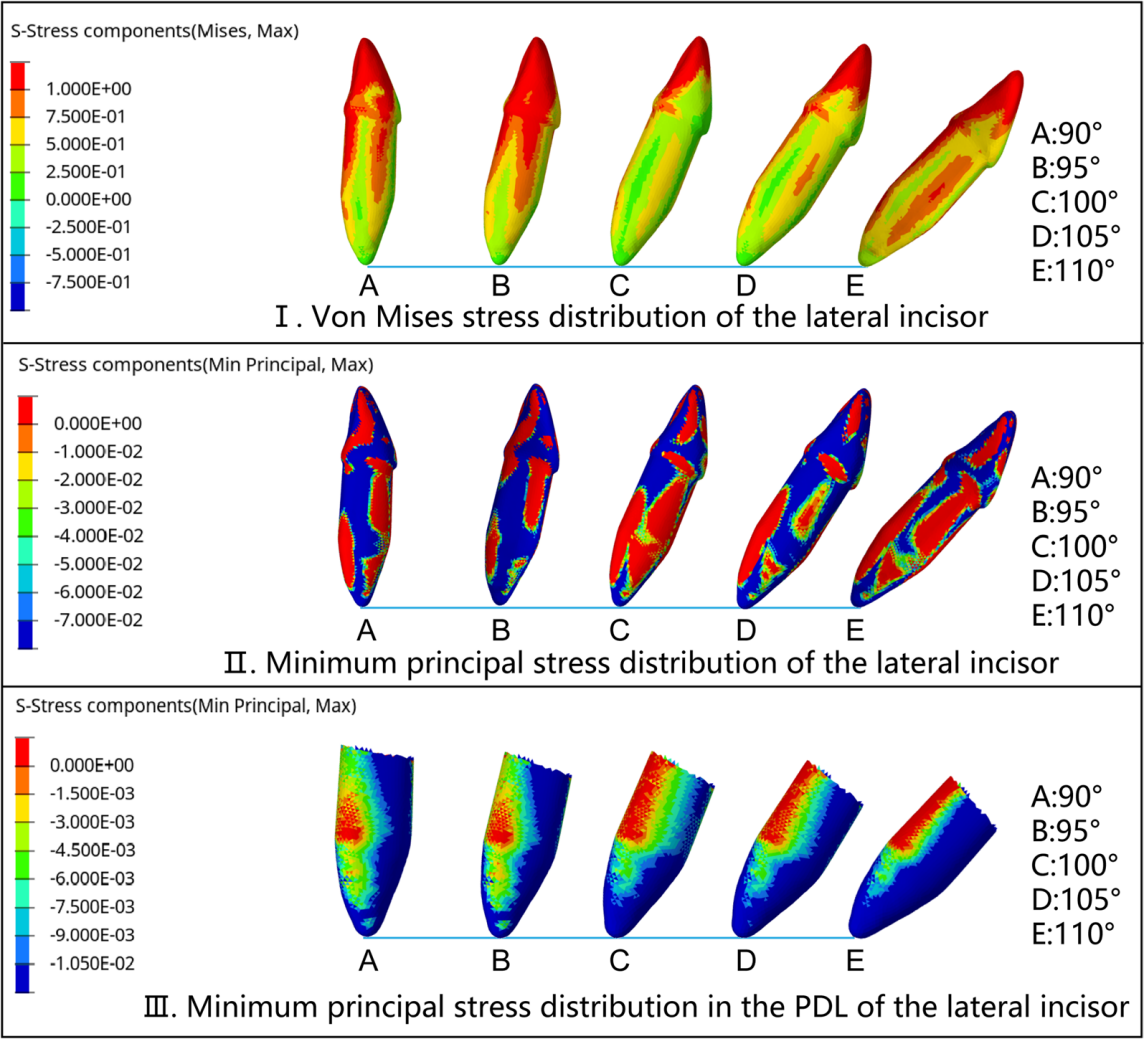
Stress distribution of lateral incisors with different IMPA during the intrusion by 0.2 mm. The sequence is A (90°), B (95°), C (100°), D (105°), and E (110°). The stress distribution of the lateral incisor was similar to that of the central incisor. **I**. The von Mises stress distribution of the lateral incisor was similar to that of the central incisor, but the average stress value was larger. **II**. The minimum principal stress distribution of the lateral incisor was similar to that of the central incisor, the value of compressive stress was smaller, and the distribution on the labial and lingual sides was more symmetrical. **III**. The minimum principal stress distribution in the PDL of the lateral incisor was concentrated on apex and labial surface, and the average compressive value was larger than that of central incisors

The von Mises stress of the incisors was mainly concentrated in the lingual fossa and incisal ridges, which were also the main contact areas with the CA. When the initial IMPA was 90° and 95°, the compressive stress on the root of the central incisor was concentrated on the apex and lingual surface. With the IMPA increased, the compressive stress concentration area changed to labial surface gradually. When the initial IMPA increased to 100°, the compressive stress area of the root was only concentrated on the root apex. When the initial IMPA was 110°, the compressive stress covered all labial areas of the root, from the apex to the root cervix. The minimum principal stress distribution of the PDLs was similar to that of the roots (Fig. 5). The stress distribution of lateral incisors was similar to that of central incisors, but von Mises stress and PDL compressive stress were larger than those of central incisors (Fig. 6).

### Force systems and stress of canines

**Fig. 7:**
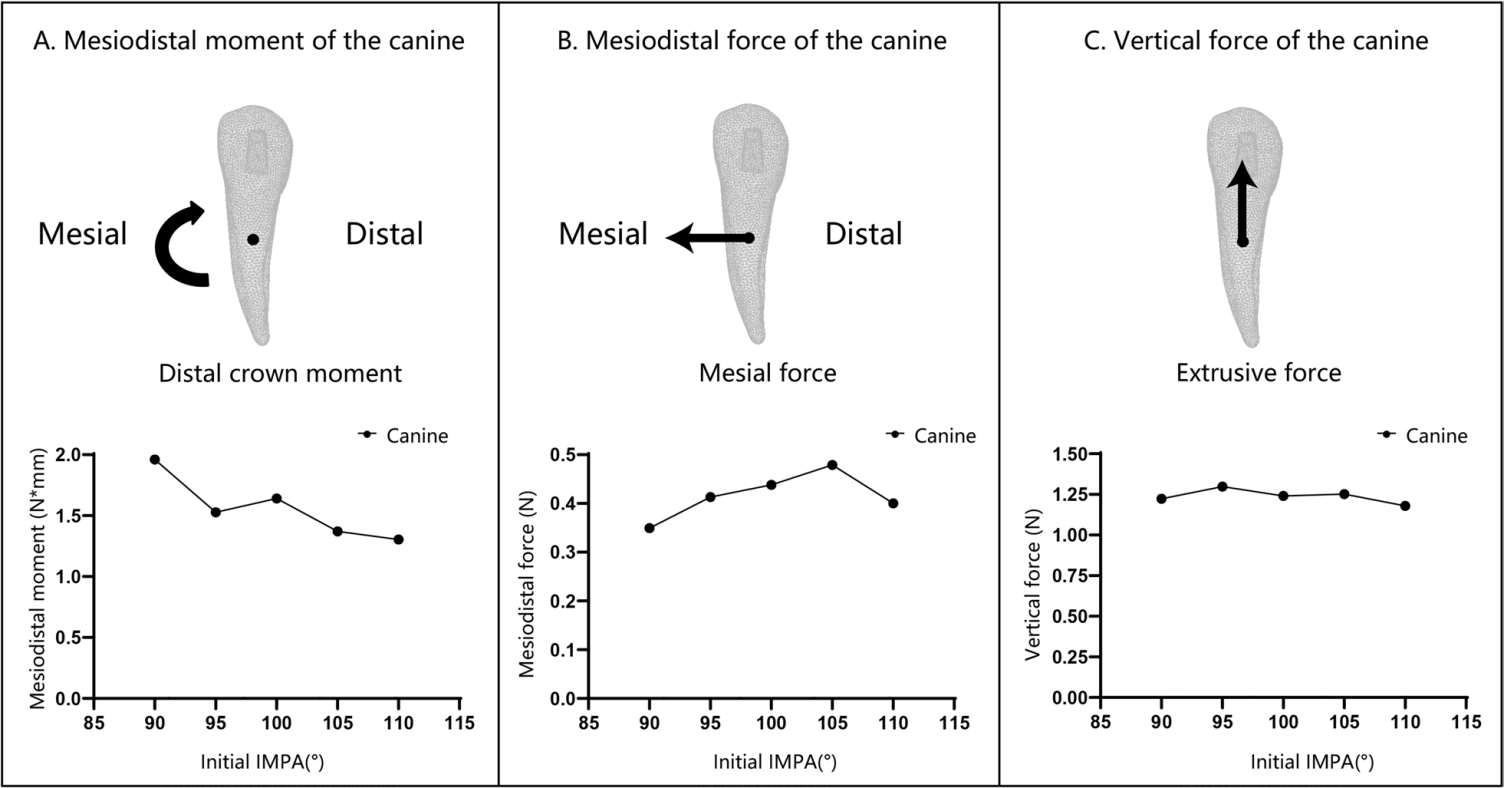
Force system analysis of the canine during the intrusion of incisors. The *X*-axis is the initial IMPA of the incisors, and the *Y*-axis is the moment and the force, respectively. The black dot in the figure is the CR, and the arrow represents the direction of the moment or force. **A**. The mesiodistal moment of the canine: The positive value represents the distal crown moment. The canine was subjected to distal crown moment. With the increase in the IMPA, the distal crown moment decreased overall. **B**. The mesiodistal force of the canine: The positive value represents the force toward the mesial direction. The CR of the canine was always subjected to the force in the mesial direction, and there was no significant change with the increase in the IMPA. **C**. The vertical force value of the canine: The positive value represents the extrusive force. The canine was subjected to an obvious extrusive force. With the increase in IMPA, the extrusive force value showed no significant change

The mandibular canines were always subjected to distal crown moment, and the CR received the force toward the mesial direction and the vertical extrusive force. With the increase in the initial IMPA of the incisors, the distal crown moment decreased slightly, and the mesiodistal and vertical force direction and value did not change significantly (Fig. 7).

**Fig. 8:**
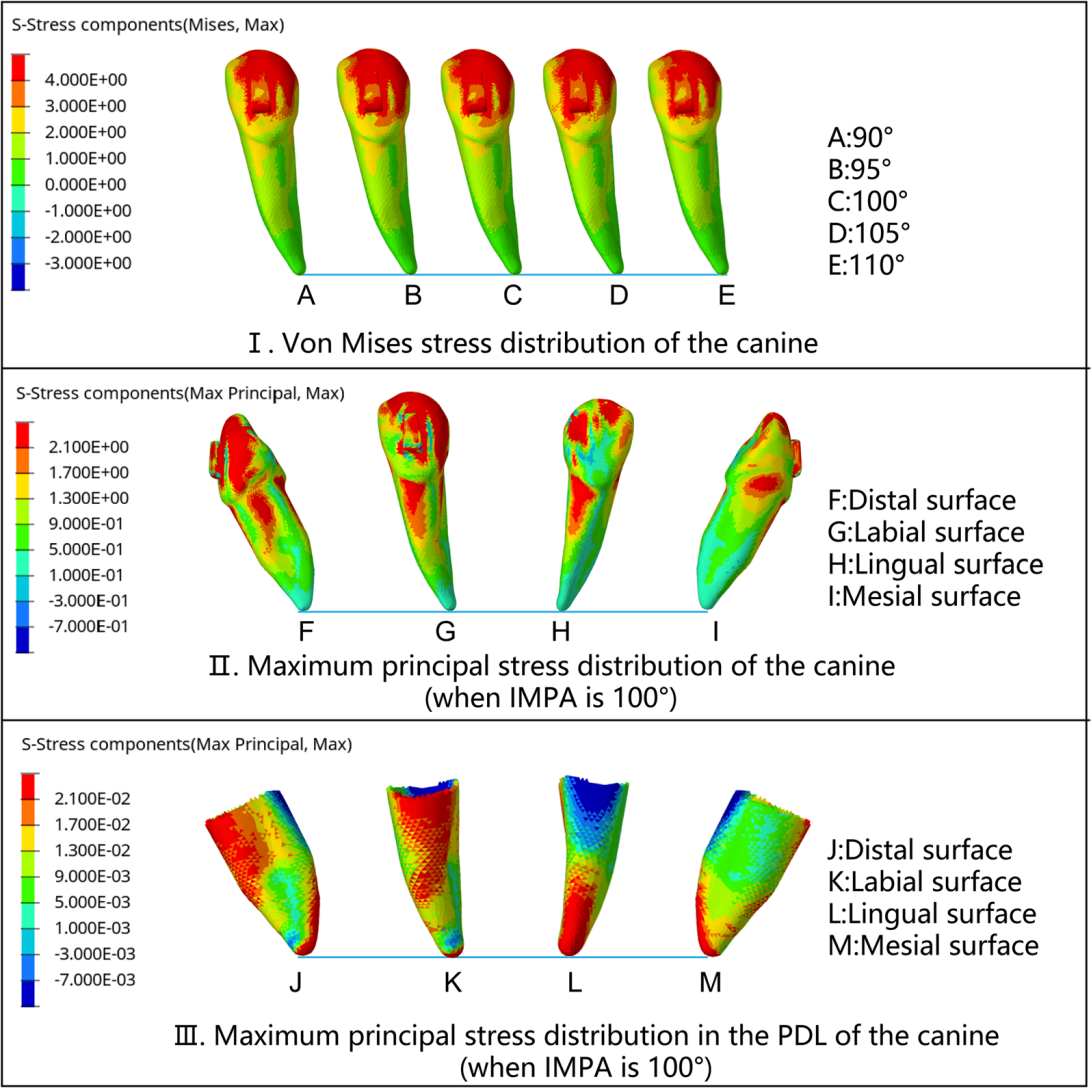
Stress distribution of the canine when the incisors were intruded by 0.2 mm. **I**. von Mises stress distribution of the canine: The stress was concentrated around the cusps, distal labial inclined planes, and attachments. The stress of the root was concentrated in the mesial cervical 1/3 area of the labial surface. With the increase in the initial IMPA, the stress did not change significantly, **II**. The maximum principal stress distribution of the canine (when the IMPA was 100°): The tensile stress was widely distributed in the cervical 1/2 of all the root surfaces. The maximum principal stress on the labial and lingual surfaces was basically symmetrical. The maximum principal stress on the distal surface was greater than that on the mesial surface. **III**. The maximum principal stress distribution in the PDL of the canine (when the IMPA is 100°): The maximum principal stress on the mesial, labial and distal surfaces was the same as that of the root. However, on the lingual surface, tensile stress was concentrated in the apical 1/2 of the PDL

The force system of the canine had no significant relationship with the change in the initial IMPA (Fig. 7), as did the stress distribution. The von Mises stress of the canines was concentrated around the cusps, distal labial inclined planes, and attachments. The maximum principal stress indicated that tensile stress was widely distributed in the cervical 1/2 of the root. The tensile stress on the labial and lingual surfaces of the root was basically symmetrical, while that on the distal surface was greater than that on the mesial surface. In PDL, the tensile stress on the mesial, labial, and distal surfaces was similar to the distribution of the root. However, on the lingual surface of the PDL, tensile stress was concentrated in the apical 1/2 of the PDL (Fig. 8). The forces generated by the CA deformation not only intruded the lower incisors but also acted on the lower canines as anchorages. This deformation force included the force toward the mesial and extrusive direction of the lower canine, while the apex tended to move labially.

### Movement trends

**Fig. 9:**
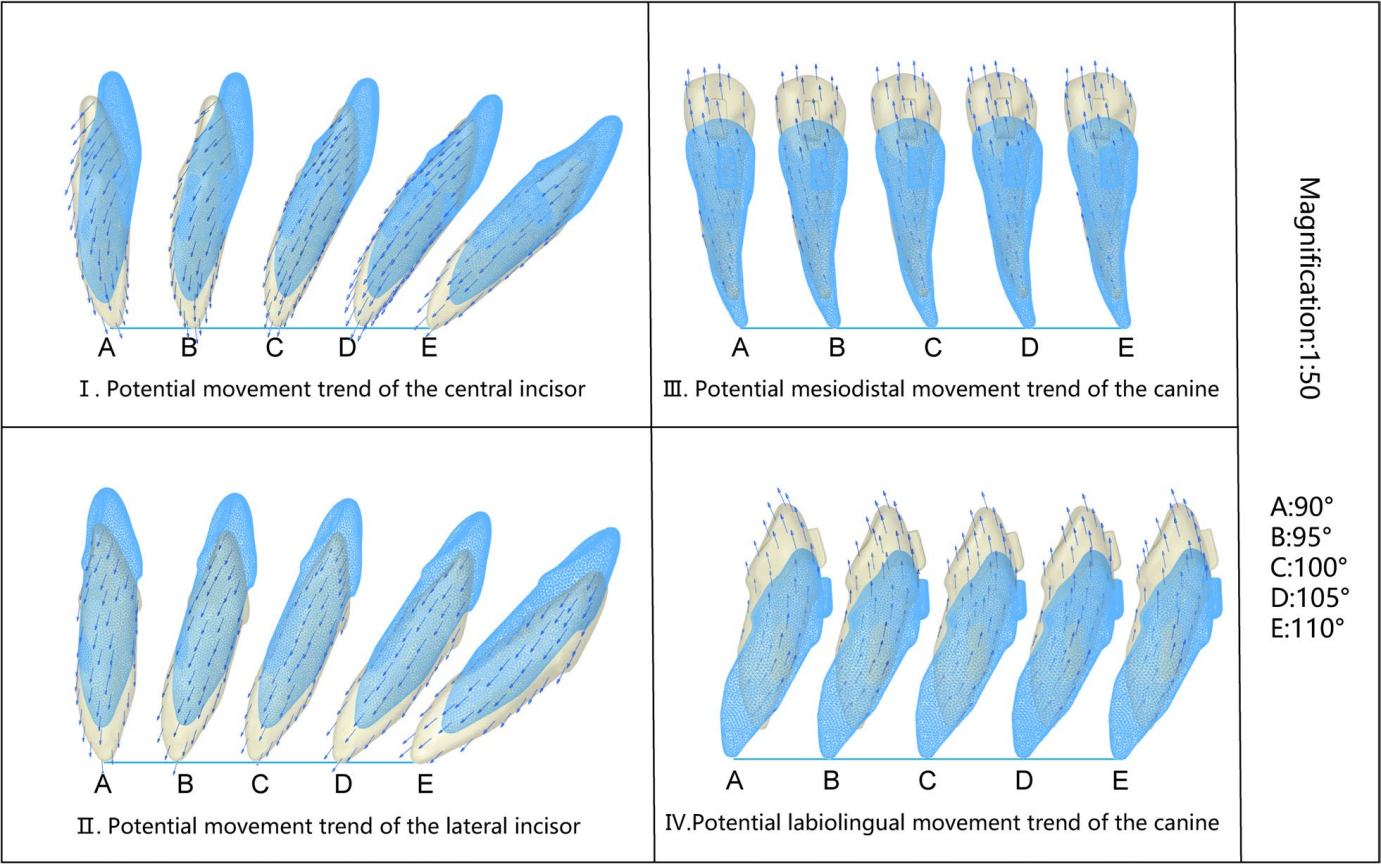
Movement trend of incisors and canines when the incisors were intruded by 0.2 mm. The sequence is A (90°), B (95°), C (100°), D (105°), and E (110°). Those in blue represent the position before the intrusion, and those in yellow represent the position after the intrusion. Blue arrows represent the direction and trend of teeth movement. The movement magnification is 50 times. **I**. Potential movement trend of the central incisor: When the initial IMPA was less than or equal to 100°, the apex rotated labially, and the crown rotated lingually, while the apex moved labially. When the IMPA was greater than or equal to 105°, the apex rotated lingually, and the crown rotated labially, while the root cervix moved labially. **II**. The potential movement trend of the lateral incisor: The movement trend of the lateral incisor was similar to that of the central incisor, but the lingual crown rotation trend was smaller and the labial crown rotation trend was more obvious. When the initial IMPA was less than or equal to 95°, the apex rotated labially, and the crown rotated lingually, while the apex moved labially. When it was greater than or equal to 100°, the apex rotated lingually, and the crown rotated labially, while the root cervix moved labially. **III**. Potential mesiodistal movement trend of the canine: The canine distal crown tipped with an extrusive trend, and the CR tended to move mesially. There was no significant change in the trend of canine movement with increasing IMPA. **IV**. Potential labiolingual movement trend of the canine: The canine crown tended to rotate lingually with the extrusion trend. There was no significant change in the movement trend with increasing IMPA

For both central and lateral incisors, when the initial IMPA was less than or equal to 95°, their apex tended to rotate to the labial side and the crown to the lingual side, while the apex moved labially. When the initial IMPA reached 100°, the potential movement direction of the central incisor remained unchanged. For the lateral incisor, when the initial IMPA reached 100°, the crown tended to rotate to the labial side, the apex to the lingual side, and the root cervix moved labially. When the initial IMPA was 105°, both the crown of the central and lateral incisors rotated labially, and the root cervix tended to move labially. The results suggested that during the intrusion, although the initial IMPA was different, the tooth root (either root apex or root cervix) always had a tendency to move to the labial side through rotation. With the increase in IMPA, the rotation direction of the teeth reversed, with direction turning points between 100° and 105° in the central incisors and between 95° and 100° in the lateral incisors. Compared with the central incisors, the lateral incisors had a greater trend of labial movement of the crown and root cervix (Fig. 9I–II). In clinical cases, when CA intrudes the lower incisors, the roots of the teeth often protrude from the labial alveolar bone, and the risk reason is unknown. The results of this paper reveal the reason for this phenomenon, which has guiding significance for the clinical application of CA.

The canine crown tended to rotate distally, the apex tended to rotate mesially, and the CR tended to move mesially. Therefore, the tendency of the root to move mesially was greater than the tendency of the crown to move mesially. In the labiolingual direction, the canine crown tended to rotate lingually. In the vertical direction, the crown tended to extrude. The movement trend of the canine did not change significantly with the increase in the initial IMPA of the incisors (Fig. 9III, IV). The results suggest that during the intrusion of incisors, the force generated by the structural deformation of the CA also caused the root of the canine to move labially and mesially, which explained the common clinical phenomenon.

## Discussion

Leveling the COS is a critical step in orthodontic treatment. The process of orthodontic treatment consists of three steps: aligning the teeth, bite opening and space closure, and fine adjustment. Therefore, for almost all orthodontic patients, the problem of intrusion of the lower incisors will be involved. Especially for patients with class II malocclusion and deep overbite, there are often different degrees of lip inclination compensation in the mandibular incisors, and the intrusion problem of the lower incisors is more arduous. Understanding the force and movement trend of the lower incisors of different IMPAs during the intrusion is a key factor in the successful treatment. CA is widely used in clinical practice, but the intrusion of lower incisors is often accompanied by unexplained root penetration from the labial alveolar bone, resulting in the risk of fenestration and dehiscence. Therefore, the mechanical study of CA intruding incisors has been a research hotspot for a long time.

3D finite element analysis is a classic biomechanical research method. The fineness of the meshing of the finite element model, the parameters of the materials, and the boundary conditions are directly related to the reliability of the simulation results. To study whether the initial biological response of tooth movement started in the alveolar bone or the PDL, Kawarizadeh et al. [15] used rat tissue sections to reconstruct a model including the right maxillary first molar, PDL, and alveolar bone. All bodies were meshed by solid elements (8 node quadrangular prism elements). The number of nodes was 8000. The results showed that the stress and strain distribution of the PDL was consistent with the distribution of osteoclasts, suggesting that the mechanical force induced by the PDL and surrounding osteoclasts was the main factor for tooth movement. To establish a complete 3D FE model of the maxillary second molar and verify its authenticity, Tajima et al. [16] used micro-CT to establish a 3D model of the isolated tooth, including pulp, dentin, and enamel, but not including PDL. The model was meshed into 10 node tetrahedral elements. The numbers of elements and nodes were 20,773 and 30,718, respectively. They compared the strain calculated by finite element analysis and the strain measured in vitro. The regression coefficient and standard error were 0.84 and 0.06, respectively, indicating that the FE model reconstructed from micro-CT data can be used as an effective model to analyze actual strain with acceptable accuracy. To study the effect of attachments on the closure of the maxillary central incisor space by the CA, Yokoi et al. [7] scanned a standard teaching model (i21D-400C, Nissin Dental Products Inc., Tokyo, Japan) by CBCT to establish a model including the maxillary dentition, PDLs, and alveolar bone. In the model, the PDL was set as a 0.2 mm uniform solid around the outer surface of the root. The teeth were divided into triangular shell elements, and the PDLs were meshed by 6-node triangular prismatic solid elements. Teeth and PDLs had a total of 67,210 units and 33,860 nodes. The results revealed that the attachments can assist the bodily movement of the central incisors. In this experiment, an industrial CT with a slice of 0.1 mm was used to scan a standard mandibular model for finite element modeling. PDLs with a uniform thickness of 0.3 mm were constructed on the roots [9]. The teeth were divided into 96,018 tetrahedral elements, and the PDLs were divided into 308,219 hexahedral elements. Existing studies have shown that in finite element analysis, the smaller the mesh element volume is, the more accurate the nonlinear continuous structure of the PDL that can be mapped, and the calculation results are more accurate [17]. Compared with the previous study, the elements of this study were divided more finely, and the quantity was larger. Compared with single-tooth modeling, the modeling of the mandibular dentition in this study more realistically reflected the interaction between teeth during intrusion. The results of this study were the stress distribution and the potential tooth movement trend during the intrusion, while the reconstruction process of the alveolar bone was not discussed. Therefore, the modeled object did not include the alveolar bone.

In this study, CA was the key force-applying tool to move the teeth, and its young’s modulus and structure should be as close as possible to the real situation. According to the existing research, at 37 °C, the elastic modulus of Invisalign is 842 ± 63 MPa, which is composed of TPU materials [18]. At 37 °C, the elastic modulus of Duran and Erkodur is 2015 ± 132.229 MPa and 1365.150 ± 262.883 MPa, respectively, both of which are composed of PTEG [19]. MC-S is a multilayer composite material, including TPU and PETG. According to ISO 527-3 [20], type 5B specimens were prepared with the material. The value of Young’s Modulus was established after performing compression tests (using an InstronH Universal Testing Machine). Finally, the Young's modulus of MC-S is 1000 MPa. In previous studies, CA was constructed as solid bodies with uniform thickness expansion along the crown shape, ranging from 0.3 to 0.75 mm [7, 21, 22]. However, in clinical practice, the thickness of the CA is nonuniform after the thermoform, and the structural characteristics of the CA have an important influence on the mechanics. Yue et al. [23] used reverse engineering technology to construct a simulated CA model with nonuniform thickness by scanning solid aligners. In this way, CA needs to be produced first and then scanned one by one to build models, which is difficult to achieve in research. Barone et al. [12] pointed out that the thickness of the CA diaphragm was 0.75 mm before thermoforming, so it is reasonable to set the CA to approximately 0.7 mm in the model after thermoforming. However, such a simulation was far from clinical reality. In the study of Gabriele et al. [8], the thickness of the Invisalign obtained by micro-CT scanning was 0.5 mm. Therefore, they expanded it uniformly along the outer surface of the crown by 0.5 mm and then smoothed it to obtain the CA model. In this paper, we used clinically applied software to simulate the thermoform and construct the structure of the CA to obtain a more realistic simulation model. Compared with the existing models, this CA model was more authentic.

The coefficient of friction (μ, the ratio of frictional force to positive pressure) between teeth and CA was an important setting for the study, but the value of μ is controversial in previous studies. In the studies of Barone et al. [12, 24] and Li et al. [25], μ was set to 0 because of the large material differences between teeth and CA and the presence of saliva as lubrication [12]. In other studies [26–30], μ was set to 0.2, which was similar to the friction coefficient between enamel and composite [26]. In the study of Goto et al. [31], μ was set to 0.5. In this study, μ was set to 0.3, which was the data obtained through the friction coefficient measurement experiment between the CA and teeth (refer to GB/T10006-2021 Plastics-Film and sheeting-Determination of the coefficients of friction).

Different patients have different inclinations of the lower incisors before treatment, which directly affects the distribution of force. Therefore, it is necessary to set appropriate IMPA groups to reflect different situations of patients with class II malocclusion. The study of Brezniak et al. [32] revealed that the average IMPA of Class II division 1 was 97.7 ± 6.4°, and the average IMPA of Class II division 2 was 97.4 ± 7.4°. The mandibular incisors of Class II malocclusion display compensatory labial inclination. Liu et al. [33], de Brito et al. [5], and Ni et al. [4] all used the upright mandibular incisor model when studying the intrusion of the mandibular incisors. To explore the effect of different IMPA on the force system and movement trend of mandibular anterior teeth during the intrusion, the initial IMPA was set to 90°, 95°, 100°, 105°, and 110° according to common compensatory inclination angles. Deep bite and deep COS can be seen in different malocclusions. Since the IMPA grouping in this research was set according to the Class II malocclusion, we mainly discuss the intrusion situation of the Class II malocclusion in the article. The results revealed that the force of the mandibular incisors was different under different initial IMPA, and the rotation direction of the teeth was reversed between 95° and 105°. In the clinical use of CA to intrude mandibular incisors, when the IMPA is less than or equal to 95°, lingual cervical bone dehiscence and labial apical bone fenestration may occur. When the IMPA was 100°, the central incisors were still at risk of apical labial penetration. For lateral incisors, labial cervical dehiscence and lingual apical fenestration may occur, while the cervical root protrudes to the labial alveolar bone. When the IMPA is 105°, both the central incisors and the lateral incisors may develop labial cervical dehiscence and lingual apical fenestration.

Different anterior tooth intrusion designs have a significant impact on the intrusion effect on mandibular incisors and canines, and the anchorage teeth always receive extrusive force. Liu et al. [33] used 3D printed CA with different designs to intrude the mandibular anterior teeth on a standard model (P12P-SB1, Nissin Dental Products Inc., Tokyo, Japan). Groups were set as a control group (G0), intrusion of canines only (G1), intrusion of incisors only (G2), intrusion of incisors and canines by the same activation (G3), and intrusion of incisors and canines by different activation (G4). In the first four designs, teeth were intruded by 0.2 mm. In G4, central incisors, lateral incisors, and canines were intruded by 0.2 mm, 0.15 mm, and 0.1 mm, respectively. Rectangular attachments were designed on the premolars and first molars, and a force sensor was used to record the vertical force value. The results showed that the canines in G2, the premolars and molars in G3, and the canines and premolars in G4 were subjected to extrusive force, that is, when the target intrusive activation was different, the adjacent teeth next to the maximum activation teeth and anchorage teeth were subjected to extrusive force. In our study, the mandibular incisors were intruded by 0.2 mm with the canines as anchors. The results showed that the canines were always subjected to extrusive force, and the central incisors and lateral incisors were subjected to intrusive force, but the intrusive force of the lateral incisors was always greater than that of the central incisors. This study also found that the canines were simultaneously subject to distal crown tip, mesial movement, and crown lingual rotation, suggesting that the root and periodontal health of anchoring teeth also needs attention in clinical practice.

Although finite element method is a classical mechanical simulation research method, it also has its limitations [34]. It is a computerized in vitro study [35] that can provide a reference for clinical practice and follow-up clinical research, but it cannot be completely equivalent to clinical trials and cannot directly guide clinical practice. Evidence at a higher level needs to be obtained through follow-up clinical trials.

## Conclusions

The result of lower incisor intrusion is obviously related to the initial IMPA. When the initial IMPA increased from 90° to 110°, the labiolingual rotation direction of the incisors reversed, but the root of the incisor always tended to tipped labially. As an anchor, the canine experienced extrusion, distal crown tip, and lingual crown rotation. It is suggested that in CA cases, root movement can still lead to alveolar bone damage despite the use of opposite moment. The results of this study explained why this phenomenon occurs. When CA is used to intrude mandibular incisors, the treatment steps should be designed according to the initial IMPA, and the structural design of CA should be improved in the future to optimize the mechanical properties to prevent the above complications.

## Data Availability

Please contact authors for data requests.

## Abbreviations

CA: Clear aligner
FE: Finite element
PDL: Periodontal ligament
IMPA: Incisor mandibular plane angles
CR: Center of resistance
COS: Curve of Spee
CBCT: Cone-beam computed tomography

## Declarations

### Concent for publication

All the participants’ informed consents were obtained.
